# In memoriam George David

**DOI:** 10.1007/s12471-018-1210-5

**Published:** 2018-11-28

**Authors:** J. J. Piek

**Affiliations:** AMC Heart Center, Amsterdam University Medical Centers, location Meibergdreef, Amsterdam, The Netherlands

Dr. George K. David passed away on September 18, 2018. He was born in Amsterdam on April 29, 1933 and lived in Hungary from 1944 to 1945. After their return to the Netherlands, his family settled in Amsterdam. In 1960, George David graduated Medical School and completed his residency training programme in cardiology at the former Wilhelmina Gasthuis (head: Professor dr. D. Durrer) in Amsterdam in 1968. In the 1960’s, cardiology became an independent subspeciality of internal medicine. At that time, professor Durrer was already a world-renowned leader in the field of electrophysiology who was eager to set up his own department of clinical and experimental cardiology at an internationally-recognised high standard. George David was appointed as staff member at this famous institution that was founded on a solid background in electrophysiology. Nevertheless, George David’s primary interest was invasive cardiology and so he served as Head of the cardiac catheterisation laboratory. At that time, the first percutaneous coronary interventions were being performed by Andreas Gruntzig in Zurich, followed by the first live courses in Zurich and Atlanta. George David was among the first participants, together with a few other Dutch cardiologists. This led to the first percutaneous coronary intervention in Amsterdam, performed by George David, together with Karel Romijn, in 1981. The developments in this field emerged rapidly and George David exhibited his skills as an excellent tutor and mentor for both residents in cardiology and fellows in interventional cardiology. He served as executive director of the Department of Cardiology (head: prof. dr. A. J. Dunning) after the Department of Clinical and Experimental Cardiology at the Wilhelmina Gasthuis was closed down and moved to the new Academic Medical Centre in Amsterdam-Zuidoost. As executive director he was responsible for the training of cardiology residents and served as a role model using his skills and in-depth knowledge in the field of cardiology and his dedicated attitude towards cardiac patients. He was a modest person and supported ambitious co-workers seeking an academic career. After his retirement in 1993, he kept in touch with those who inspired him the most. He experienced a happy period, together with Marianne, living at their beloved house at the Koningslaan in Amsterdam overlooking the Vondelpark.

**Figure 1:**
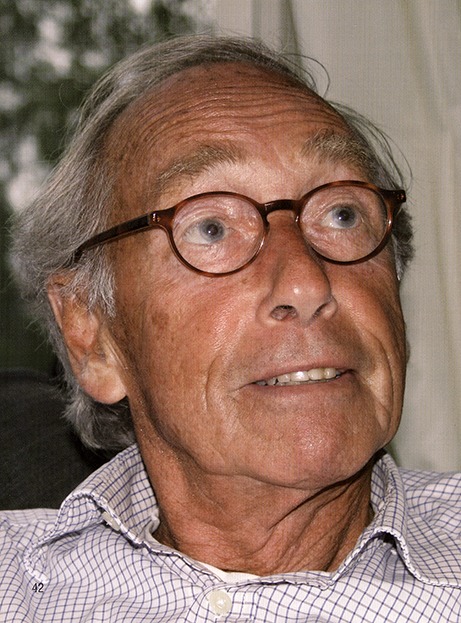
Dr. George K. David

He suffered from neuropathy. Marianne passed away in 2009 and when his disease progressed he moved to a nursing home where he spent the last two years of his life. His mental health remained intact.

He passed away in the presence of his family and loved ones. We will remember him as an éminence grise in the field of cardiology and as a person who can be best characterised as every inch a gentleman.

